# Supplementary material for: Saccharomyces boulardii (CNCM I-745) improves intestinal damage in sepsis by remodeling intestinal flora structure
Source: Front Cell Infect Microbiol. 2025 Jul 31;15:1602792. doi: 10.3389/fcimb.2025.1602792 (PMC12350329; doi:10.3389/fcimb.2025.1602792)
Supplement: Supplementary file 1 [file DataSheet1.zip › summary/summary/8_Advanced_analyse/2_PICRUSt2_result/KEGG/Group.DW4_vs_CW4_vs_BW4_vs_AW4/PICRUSt2_KEGG_level2_Group.DW4_vs_CW4_vs_BW4_vs_AW4_diff.pdf]

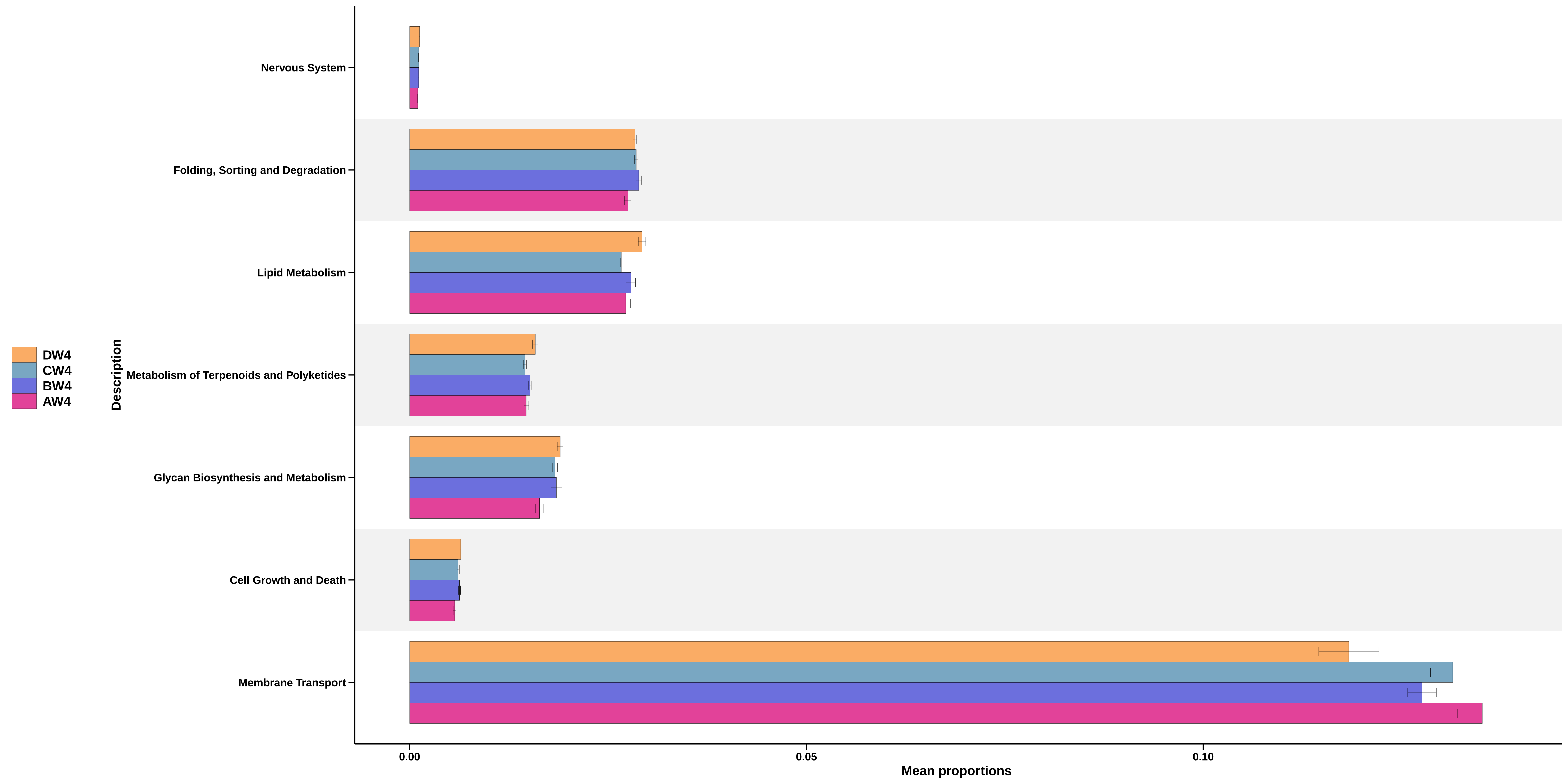
DW4
CW4
BW4
AW4
Description
Nervous System
Folding, Sorting and Degradation
Lipid Metabolism
Metabolism of Terpenoids and Polyketides
Glycan Biosynthesis and Metabolism
Cell Growth and Death
Membrane Transport
0.00
0.05
0.10
Mean proportions
